# Novel Precursor-Derived Meso-/Macroporous TiO_2_/SiOC Nanocomposites with Highly Stable Anatase Nanophase Providing Visible Light Photocatalytic Activity and Superior Adsorption of Organic Dyes

**DOI:** 10.3390/ma11030362

**Published:** 2018-03-01

**Authors:** Eranezhuth Wasan Awin, Abhijeet Lale, Kollamala Chellappan Nair Hari Kumar, Umit Bilge Demirci, Samuel Bernard, Ravi Kumar

**Affiliations:** 1 Laboratory for High Performance Ceramics, Department of Metallurgical and Materials Engineering, Indian Institute of Technology Madras (IIT Madras), Chennai 600036, India; eranezhuth@gmail.com (E.W.A.); kchkumar@iitm.ac.in (K.C.N.H.K.); 2 Science des Procédés Céramiques et de Traitements de Surface (SPCTS), UMR CNRS 7315, Centre Européen de la Céramique, 12 rue Atlantis, 87068 Limoges CEDEX, France; abhijeet.lale@unilim.fr (A.L.); samuel.bernard@unilim.fr (S.B.); 3 IEM (Institut Europeen des Membranes), UMR 5635 (CNRS-ENSCM-UM), Universite Montpellier, Place E. Bataillon, F-34095 Montpellier, France; Umit.Demirci@iemm.univ-montp2.fr

**Keywords:** silicon oxycarbide, precursor derived ceramics, photocatalysis, adsorption

## Abstract

Titania (TiO_2_) is considered to have immense potential as a photocatalyst, the anatase phase in particular. There have been numerous attempts to push the limits of its catalytic activity to higher wavelengths to harness the visible electromagnetic radiation. Most of the investigations till date have been restricted to fine-tuning the bandgap by doping, control of defect chemistry at the surface and several to first principle simulations either with limited success or success at the cost of complexities in processing. Here, we report a simple and elegant way of preparing ceramics through precursor chemistry which involves synthesis of macroporous and mesoporous nanocomposites with in situ formation of TiO_2_ nanocrystals into a robust and protecting SiOC matrix. The in situ nanoscaled TiO_2_ is anatase of size 9–10 nm, which is uniformly distributed in an amorphous SiOC matrix forming a new generation of nanocomposites that combine the robustness, structural stability and durability of the SiOC matrix while achieving nanoscaled TiO_2_ functionalities. The stabilization of the anatase phase even at temperature as high as 1200 °C was evident. With an average pore size of 6.8 nm, surface area of 129 m^2^/g (BET) and pore volume of 0.22 cm^3^/g (BET), mesoporosity was achieved in the nanocomposites. The composites exhibited visible light photocatalytic activity, which is attributed to the Ti–O–C/TiC bonds resulting in the reduction of band gap by 0.2 to 0.9 eV. Furthermore, the heterojunction formed between the amorphous SiOC and crystalline TiO_2_ is also expected to minimize the recombination rate of electron-hole pair, making these novel nanocomposites based on TiO_2_ extremely active in visible wavelength regime.

## 1. Introduction

Silicon oxycarbide (SiOC), known for its high temperature stability, is a network-like structure consisting of an amorphous SiOC phase and free carbon [1]. Owing to the high mechanical stability, chemical inertness and oxidation resistance, SiOC offers to be a promising candidate as a host to anchor the photocatalyst [2,3,4,5]. Recently, incorporation of titanium into Si–O–C has shown to modify the crystallite state of this multiphasic material and is expected to offer photocatalytic activity depending up on the formation of catalytically active phases [6]. 

Titania has been widely investigated as a photocatalyst for the degradation of contaminants ever since its photocatalytic effect was reported by Fujishima and Honda in 1972 [7]. Extensive studies have been carried out in the areas of water purification, air purification and solar cells because of its low cost and high thermal stability [8,9,10,11,12]. The kinetically stabilized anatase phase is more photocatalytically active which transforms to the thermodynamically stable rutile phase on heating [13,14]. However, the photoexcitation in the UV-region and high recombination rate of the photogenerated electron-hole pair limits its applications for practical purposes. Another drawback is the poor adsorption of titania to some of the organic pollutants [15]. Several methods have been adopted in the past so as to excite TiO_2_ in the visible light, which includes non-metal doping [16,17,18,19,20], non-metal co-doping [21,22], oxygen-rich TiO_2_ modification [23], noble and transition metal deposition [24,25,26,27,28,29,30,31,32,33], dye sensitization [34,35,36], coupled semiconductors [37,38] and defect induced doping [24]. 

In general, low concentration of dye can only be removed by the photocatalytic degradation process. Hence, the failure to treat highly concentrated dye solution has necessitated the need to develop materials exhibiting a combinatorial effect of high adsorption and photocatalysis. Immobilization of TiO_2_ on a mesoporous host with high surface area can immensely contribute to the enhancement in photocatalytic activity [39,40,41]. The photocatalytic activity of TiO_2_/silicon carbide (SiC) systems reported by Christian et al. has been attributed to the synergetic effect between both the semiconductors that reduces the recombination rate of the electron–hole pair [42]. TiO_2_ coated on three-dimensional β-SiC foam, under UV-radiation was found to photodegrade [43]. In addition to this, the formation of heterojunction has been found to effectively decrease the recombination rate of photogenerated electron–hole pairs [44,45,46]. Recently, Hojamberdiev et al. demonstrated the photocatalytic activity of mesoporous SiOC/TiO_2_ and SiOC/N-doped TiO_2_ in which TiO_2_ particles were added ex situ [47]. However, the ex situ addition of titania particles in an amorphous matrix inherently leads to problems such as agglomeration and large grain growth [48]. In addition, the photocatalytic effect of a material depends on its crystallinity, morphology and specific surface area [49,50,51,52]. Hence, the processing route plays a major role.

The precursor derived ceramic (PDC) route allows enough flexibility in fine tuning the composition as well as the micro/nanostructures of the composites [53,54,55,56]. The low processing temperature and the capability to produce intricate shape makes it unique among the processing routes. This route is well adapted to prepare nanocomposites through an in situ process as we target here. The basis for this approach comes from the reaction between two precursors in which uniform chemical composition is established at molecular scale. The resulting compound is converted into a single-phase amorphous ceramic in the first pyrolysis step. The latter is subsequently heat-treated at higher temperature to initiate the crystallization and provide the material with tuned phase composition and nano-/microstructure organization [57,58,59,60,61]. The present work investigates a precursor approach towards the in situ synthesis of nanocrystalline TiO_2_ in a highly thermally robust amorphous SiOC matrix. The resulting nanocomposites show that the presence of carbon in the TiO_2_ lattice allows harnessing visible light for photocatalytic activity. The anatase phase stability was greatly enhanced (up to 1200 °C) in the macroporous composite, attributable to the presence of an amorphous SiOC matrix. Furthermore, the confinement of carbon doped TiO_2_ nanocrystals in a thermally stable amorphous matrix is expected to avoid their agglomeration and grain growth.

In order to synthesize mesoporous nanocomposite, hard templating approach was adopted resulting in enhanced surface area and adsorption kinetics. Introduction of mesoporosity in SiOC assists in high adsorption and eventually visible light photocatalysis is achieved by the presence of the stabilized anatase phase. The differences in synthesis route adopted for the production of macroporous and mesoporous nanocomposite was detailed and the resulting phase, crystal size, bonding characteristics, porosity, micro and nanostructural features were compared. The possible mechanism of adsorption and photocatalysis is detailed from the methylene blue dye degradation studies.

## 2. Experiment

### 2.1. Design of Meso-/Macroporous Compounds

#### 2.1.1. Synthesis of Macroporous Nanocomposite

**Figure 1:**
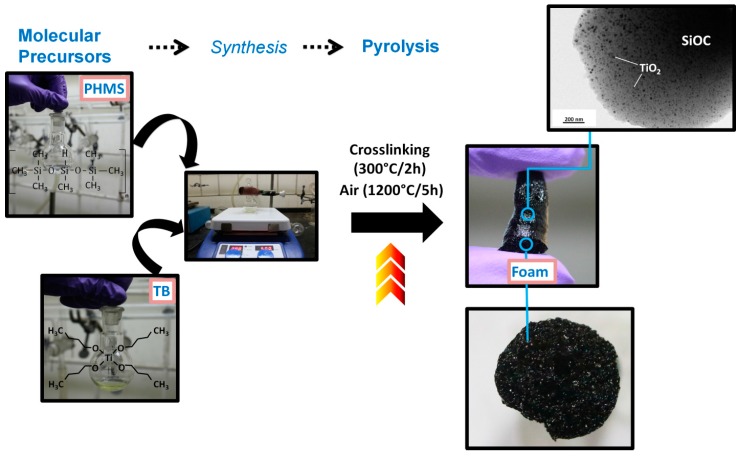
Schematic diagram of the general process for the synthesis of macroporous TiO_2_/SiOC nanocomposite.

The precursors selected for the synthesis of TiO_2_/SiOC were commercially available polyhydridomethylsiloxane (PHMS) and titanium n-tetrabutoxide (TB) (Sigma-Aldrich, Bangalore, India). The liquid precursors were chosen for facile processing and equal vol % of the precursors were taken in a round bottom flask, stirred for 60 min continuously using a magnetic stirrer to initiate the reaction. The mixed solution was then transferred to a tubular furnace and was cross-linked at 300 °C for 2 h. The cross-linked polymer was then subsequently heat-treated to 1200 °C for 5 h with a heating rate of 5 °C/min in air. A foamy structure was produced which was ground using a mortar and pestle to a fine powder. The schematic of the synthesis procedure is shown in Figure 1. The thermogravimetric analysis (Figure S1) for a mixture of 50 vol % of each of the precursors (carried out in air) suggests that the initial polymeric mixture exhibited mass loss in three stages. The first stage (25–250 °C) is assigned to the loss of water and alcohols whereas the second (250–450 °C) and third stages (450–800 °C) could be attributed to the decomposition of the polymer and release of methane and hydrogen gases, respectively. There was no noticeable mass loss beyond 800 °C. The crosslinking temperature was chosen to be 300 °C to maximize the yield.

#### 2.1.2. Synthesis of Mesoporous Nanocomposites

**Figure 2:**
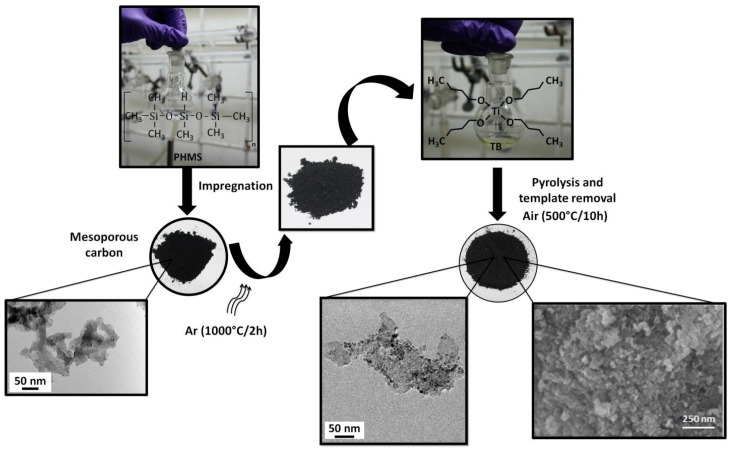
Schematic diagram illustrating the generalized process for the synthesis of mesoporous TiO_2_/SiOC nanocomposite.

The first stage of synthesis involves impregnation of 1 mL of PHMS into 0.2 g of mesoporous carbon (Sigma-Aldrich, Bangalore, India) by continuously stirring for 48 h. The mixture was then pyrolyzed in argon atmosphere at 1000 °C for 2 h maintaining a heating rate of 2 °C/min in ambient conditions. The mixing of pyrolyzed product with 1 mL of TB for 24 h and subsequently heat-treated at 500 °C in air for 10 h forms the second stage. The completion of the first stage involves the formation of mesoporous SiOC–C composite and the second stage results in the materialization of TiO_2_/SiOC nanocomposite removing the carbon template. The schematic of the synthesis procedure is shown in Figure 2. The disordered mesoporous nature of the nanocomposite was visualized through SEM and the pore size estimated using Brunauer-Emmett-Teller (BET) technique was found to be around 6.8 nm.

### 2.2. Characterizations

The thermogravimetry analysis (NETZSCH STA 409, Selb, Germany) of initial polymeric mixture [PHMS and TB (1:1)] was carried out in static air flow at a heating rate of 5 °C/min to 1400 °C. The Fourier-transform infrared spectroscopy (FTIR) analysis (Perkin Elmer Spectrum, Hatfield, PA, USA) of the powder sample was also done to ensure whether proper ceramization has occurred. The samples were mixed with infra-red IR irradiated potassium bromide and pelletized before subjecting to FTIR analysis. The samples were subjected to XRD (D8 Discover, Bruker AXS X-ray diffractometer, Madison, WI, USA), scan limit in the range 20° ≤ 2θ ≥ 90° in increments of 0.02° with a time per step of 15 s. The peaks were identified with the aid of Joint Committee on Powder Diffraction Standards (JCPDS) data files for TiO_2_. The average crystallite size was determined using Scherrer’s equation assuming the shape of the particles to be spherical. The Rietveld analysis of the diffracted pattern was performed using MAUD software. The software applies RITA/RISTA method for the analysis which was developed by Wenk, Ferrari and Lutterotti [62]. The Raman spectra (Labram HR 800, Horiba, Minami-Ku, Kyoto, Japan) of the powdered samples was acquired with a He-Ne laser source (488 nm), recorded in the range between 100 and 4000 cm^−1^. The microstructural as well as elemental analysis of the sample was done using scanning electron microscopy (SEM; FEI Quanta 200 & 400, Calabasas, CA, USA) and Energy-dispersive X-ray spectroscopy (EDS) analysis (FEI Quanta 200 & 400, Calabasas, CA, USA). The samples were subjected to gold sputtering prior to the SEM analysis. The nano-structural features of the samples (particle size and crystal structure) was ensured using transmission electron microscopy (JEOL 3010, Akishima, Tokyo, Japan) operating at 200 kV accelerating voltage. The powder sample was dissolved in acetone, ultrasonicated for 1 h and a droplet was poured on to a copper grid for transmission electron microscopy (TEM) analysis. The X-ray photoelectron spectroscopy XPS (Omnicron nanotechnology, ESCA-14, Taunusstein, Germany) investigation was carried out at ambient atmosphere maintaining the base pressure in the measurement chamber to less than 10^−7^ Pa. Al Kα (1486.6 eV) was used as radiation source and CASA XPS software was used to deconvolute the XPS spectra. The surface area (S_BET_) as well as the pore volume was determined using a BET analyzer (TriStar 3020, Micromeritics, Norcross, GA, USA). The N_2_ adsorption-desorption isotherm at −196 °C was used to quantify the porosity. Prior to the measurement, the powder was preheated at 150 °C for 12 h in vacuum.

### 2.3. Photocatalytic Studies

The reaction was carried out in a photoreactor (Heber scientific HIPR-MP400, Tamil Nadu, India). The dye, methylene blue (100 mL, 0.03 mM) was taken in a glass vessel to which 50 mg of the catalyst was added. The solution was then placed on a magnetic stirrer, which was continuously stirred for 2 h in the dark in order to attain the adsorption–desorption equilibrium. Subsequently, the solution was exposed to visible light radiation (500 W tungsten halogen lamp, with spectral distribution from 400–700 nm) at ambient conditions and under vigorous stirring. A cut off liquid filter (NaNO_2_) was used to eliminate any traces of UV light from the tungsten lamp. The degradation study of the dye was carried out with the aid of UV-Vis spectrophotometer (ThermoFisher Scientific, Evolution 220, Waltham, MA, USA). 3 mL was collected at required time intervals (0, 30, 60, 120, 150, and 180 min) and was centrifuged to eliminate the solid particles. The band gap of the sample was determined using UV−vis diffuse reflectance spectroscopy (UV−vis diffuse reflectance spectroscopy DRS) with an integrated sphere attachment.

## 3. Results and Discussion

### 3.1. Phase Evolution

**Figure 3:**
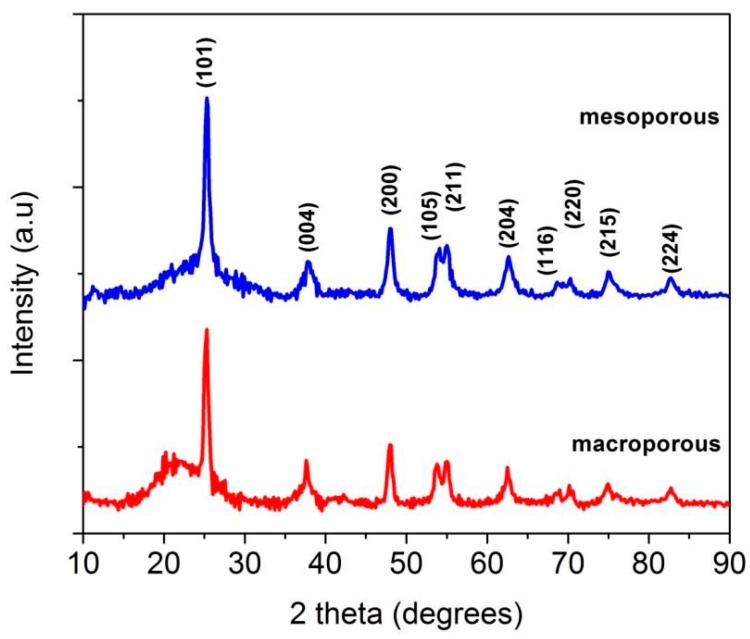
XRD of macroporous and mesoporous nanocomposites revealing the presence of anatase phase in an amorphous SiOC matrix (JCPDS card No. 20-2242).

X-ray diffractograms (XRD) of macroporous and mesoporous materials showed in Figure 3 were used to understand the phase evolution of the nanocomposites. The XRD results clearly indicate the presence of anatase phase (JCPDS card No. 20-2242). Typically, the transformation temperature in undoped TiO_2_ from anatase to rutile is in the temperature range of 700–800 °C [63]. However, here, the anatase phase is stable till 1200 °C for macroporous sample which is attributed to the presence of the amorphous matrix that hindered the conversion from anatase to rutile phases. In the case of macroporous nanocomposite, the temperature was confined to 1200 °C since increasing the temperature above 1200 °C resulted in the crystallization of the undesirable rutile and silica phases (Figure S2), which are photocatalytically not as active as anatase [64]. The average crystallite size determined using Scherrer’s equation from the full width half maxima (FWHM) of the most intense TiO_2_ peak (101), was found to be 9 and 11 nm for macroporous and mesoporous materials, respectively. 

The effect of varying volume fraction of the initial precursors on the crystallization of the phases present in the macroporous samples is displayed in Figure S3. It is clear that crystallization of anatase phase occurred only at 50 vol % of TB, whereas at 10 and 30 vol % the material remained predominantly amorphous at 1200 °C. This is most probably due to increased diffusion barriers offered by the thermally stable Si–O–C matrix [54]. Further increase in amount of TB resulted in the crystallization of rutile phase. Hence, 50 vol % was considered to be optimum. The Rietveld refinement of the diffractograms of macroporous and mesoporous nanocomposites (Figure 3) using Material Analysis Using Diffraction MAUD indicated the crystalline anatase phase to be ~34 wt % and 35 wt %, respectively and the rest being amorphous (Figure S4).

**Figure 4:**
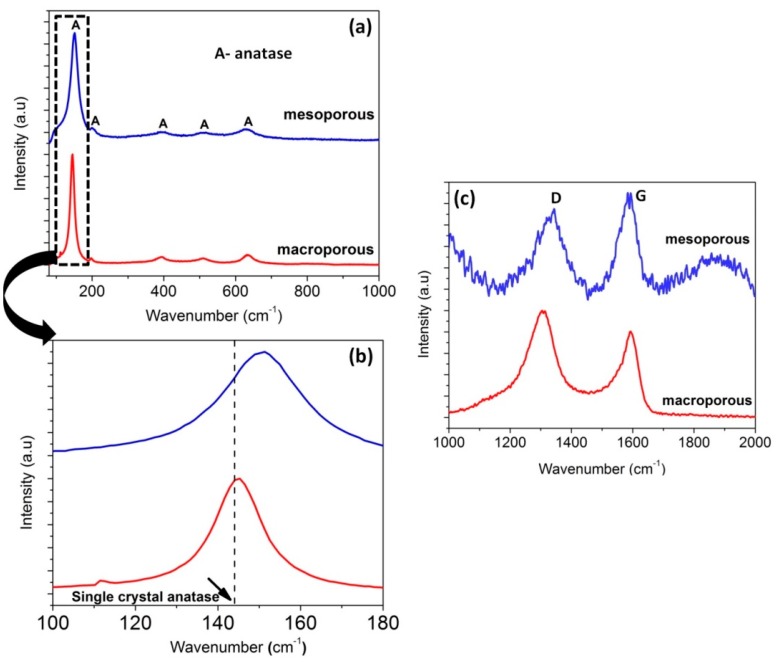
Raman spectra (**a**) of the samples revealing the anatase phase of TiO_2_ in macroporous and mesoporous nanocomposites (**b**) exemplifying the shift in peak and (**c**) revealing the presence of free carbon.

Raman spectroscopy performed on the as-synthesized samples confirmed the presence of anatase and free carbon (graphitic). The spectra shown in Figure 4a indicate the E_g_, B_1g_, A_1g_ or B_1g_, and E_g_ modes of the anatase phase. It confirms the absence of rutile phase in the samples. The Raman features of single crystal anatase as well as the macroporous and mesoporous nanocomposites are tabulated and shown in Table S1. However, in comparison with the single crystal anatase, both macroporous and mesoporous samples exhibited a frequency shift for E_g_ peak at 144 and 639 cm^−1^ (blue shift) whereas a red shift was observed for B_1g_ (399) and A_1g_ (519) peaks (Figure 4b). The shift in peaks could be attributed to the lower crystallite size and oxygen deficiency [65]. The crystallite size calculated from Scherrer’s equation also substantiates the frequency shift due to phonon confinement. Since the macroporous and mesoporous nanocomposites were synthesized via precursor route and involved heat-treatment, non-stoichiometry is expected [66]. 

It is well known that the majority of Si-based carbide/carbonitride/oxycarbide ceramics prepared from preceramic polymers are composed of free carbon at elevated temperature. The two bands at ~1300–1360 and ~1580–1600 cm^−1^ represents the D (disordered) and G (graphite) peaks, respectively and correspond to the defects present in the hexagonal graphitic structure (Figure 4c) [67].

### 3.2. Microstructural and Nanostructural Features

**Figure 5:**
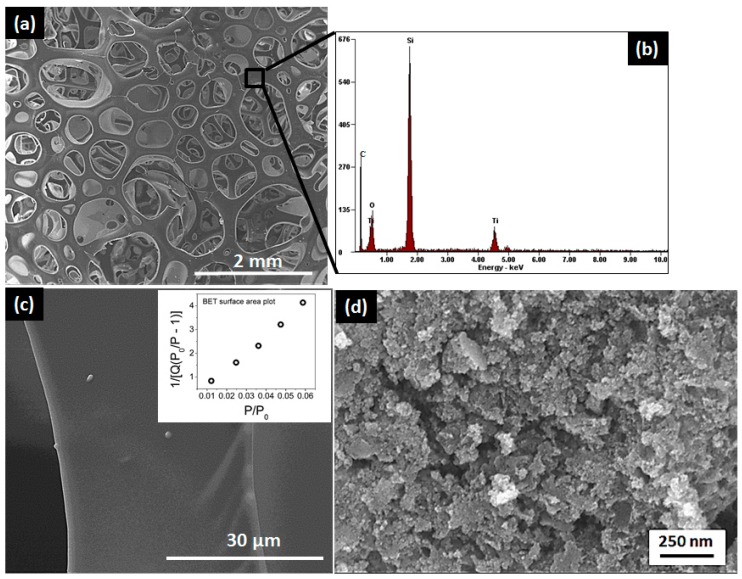
(**a**) SEM micrograph revealing the foamy nature of the macroporous sample; (**b**) corresponding EDS analysis; (**c**) Higher magnification micrograph, inset: BET surface area plot; (**d**) micrograph of mesoporous sample revealing disordered mesoporous structure.

The microstructural characterization carried out with the aid of SEM revealed a foamy appearance for the macroporous samples (Figure 5a). The EDS analysis (Figure 5b) confirmed the presence of silicon, titanium, oxygen and carbon in the nanocomposite. The micrographs of the strut at higher magnification (Figure 5c) confirmed the absence of mesopores, which were as expected since the heat-treatment temperature chosen in the synthesis of macroporous samples (1200 °C) resulted in the sintering of particles leading to the collapse of pores [68]. The micrograph of mesoporous samples as shown in Figure 5d, indicates particle agglomeration with disordered mesoporous structure. The SEM and TEM micrographs of mesoporous carbon are shown in Figure S5.

**Figure 6:**
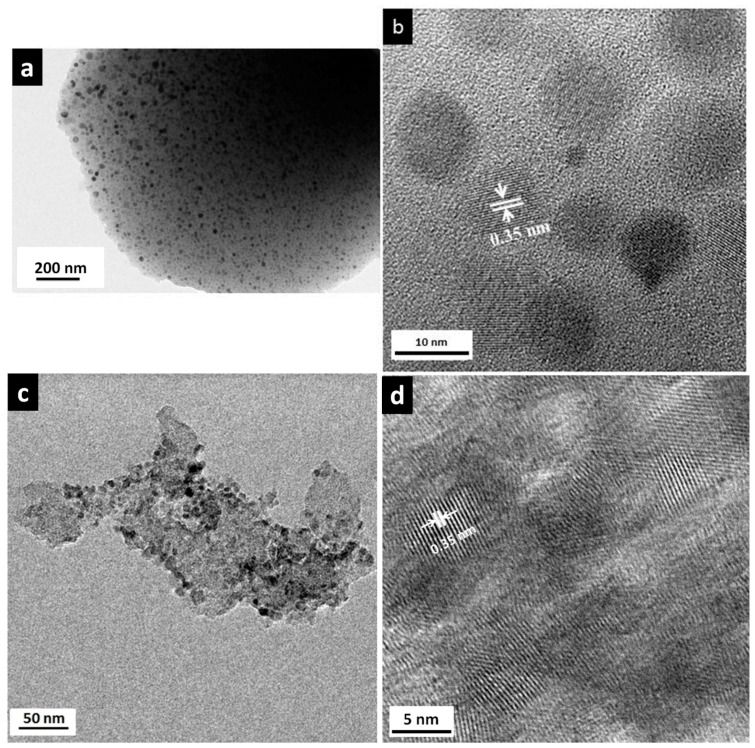
(**a**,**c**) TEM micrographs of macroporous and mesoporous nanocomposites revealing the well dispersed TiO_2_ nanoparticles in an amorphous SiOC matrix, respectively; (**b**,**d**) lattice fringes confirming the presence of anatase phase in macroporous and mesoporous nanocomposites, respectively.

The TEM investigation of macroporous and mesoporous nanocomposites shown in Figure 6 confirmed the nanocomposite structure of the materials. 

The TEM micrographs confirmed the XRD results of the same sample. It indicates that a phase segregation of TiO_2_ homogeneously occurs in the samples. The macroporous and mesoporous specimens consist of homogeneously dispersed small nuclei embedded in an amorphous matrix. The size of the nanocrystals varies from 9 to 12 nm, which is in agreement with the size measured from XRD. Although the pyrolysis temperature is relatively high, high resolution transmission electron micrographs HRTEM highlight the interest of our approach to prevent TiO_2_ coarsening and retain the anatase nanophase.

The well-defined interplanar spacing was measured from Figure 6b,d; it was found to be 0.35 nm and can be assigned to the (101) plane of anatase. The indexing of selected area electron diffraction (SAED) pattern (Figure S6a,b) confirmed the TiO_2_ anatase phase as substantiated by the XRD. The SAED pattern exhibited a discontinuous ring like pattern with distinct spots which indicates the random orientation of TiO_2_ crystals. The SiOC matrix exhibits a diffuse ring pattern (not shown), which is in accordance with the amorphous nature of the matrix. 

### 3.3. Characterization of Nanocomposites at the Meso-/Macroscopic Scales

**Figure 7:**
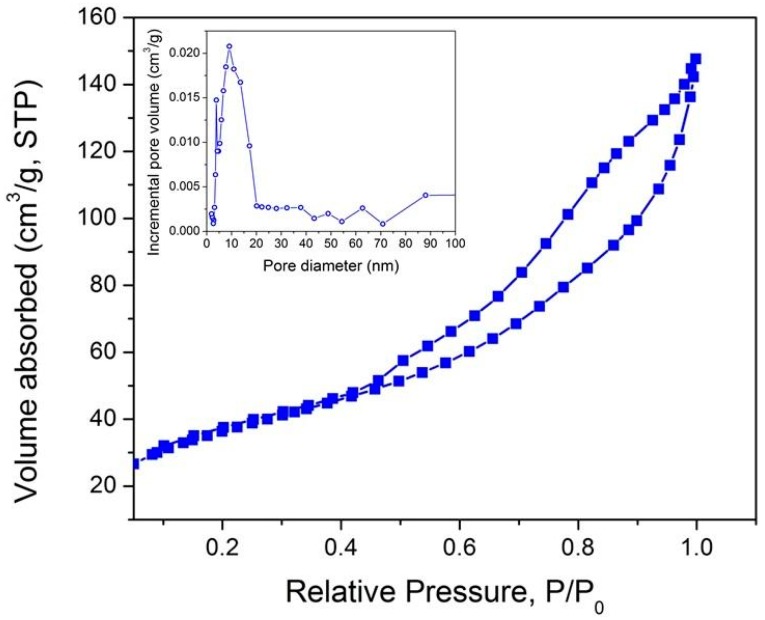
Nitrogen adsorption-desorption curve of the mesoporous sample at −196 °C (inset: Pore size distribution curve).

The pore architecture of the macroporous and mesoporous samples was assessed at the mesoscopic length scale by nitrogen gas adsorption–desorption measurements at 77 K. The BET surface area of the macroporous nanocomposite was found to be very small, 0.06 m^2^·g^−1^ (not shown) as expected. The N_2_ adsorption-desorption curve of the mesoporous sample is shown in Figure 7.

Based on IUPAC classification [69,70], the adsorption–desorption isotherms of the mesoporous sample revealed a type IV-curve suggesting that the samples have uniform mesoporous channels. The shape of the isotherms is asymmetrical with a desorption branch steeper than the adsorption branch at a relative pressure (*P*/*Po*) from 0.45 to 0.95 indicative of H2 hysteresis loops which are generally found in disordered porous materials or in ordered mesoporous material with 3-D cage-like pores and interconnected pores [71,72]. Therefore, we can say that the sample is mainly mesoporous bearing interconnected porosity. A specific BET surface area of 129 m^2^·g^−1^ is measured. 

The total pore volume determined from the amount of nitrogen adsorbed at P/Po = 0.97 is 0.22 cm^3^·g^−1^. The pore size distribution (PSD) calculated from the desorption branch by means of the Barett–Joyner–Halenda method is centered at 6.8 nm. 

**Figure 8:**
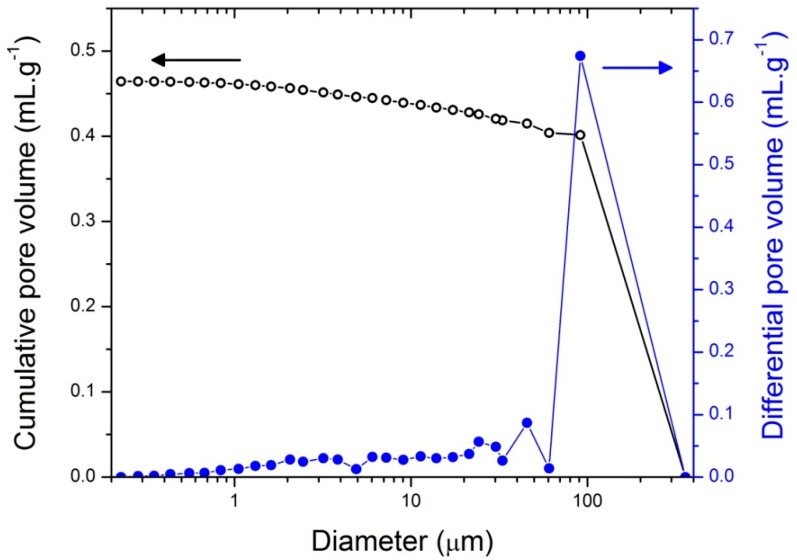
Pore size distribution of macroporous nanocomposites by mercury intrusion porosimetry.

In order to qualify and quantify the macroporosity, mercury intrusion porosimetry was investigated on the macroporous nanocomposite (Figure 8). Here, we have to specify that mercury porosimetry provides information only on the features that control the mercury intrusion—the windows that connect adjacent macropores—and not on the macropore diameters themselves. The first important feature is that the macroporous nanocomposite is robust enough to endure mercury impregnation without collapsing. The sample exhibits porosity as high as 53 vol % and an intrusion volume of 0.46 cm^3^·g^−1^. 

The macroporous nanocomposite displays a bulk density as low as 1.15 g·cm^−3^. The foam exhibited a macroporous behavior with mean diameters centered at ~25 µm, 45 µm and ~90 µm. 

### 3.4. Adsorption/Photocatalyic Degradation Studies

**Figure 9:**
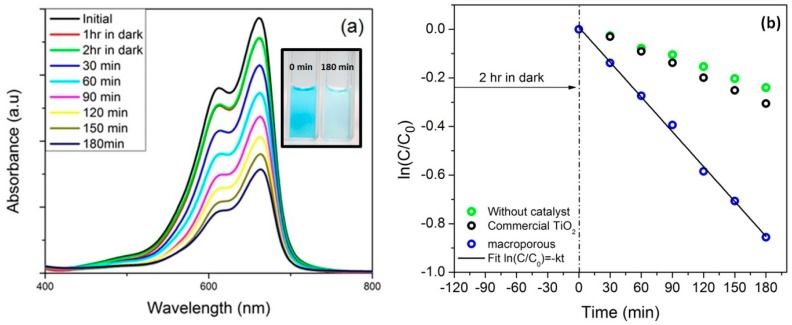
(**a**) Time dependent UV-vis absorption spectra of macroporous nanocomposite under visible light (inset: Photograph revealing the decolorization of MB); (**b**) kinetic curves of the degradation of MB by macroporous nanocomposite.

The adsorption/photocatalytic activity of macroporous nanocomposites was evaluated by measuring the decrease in concentration of methylene blue dye (MB) in dark as well as under visible light. The photocatalytic efficiency of the samples was measured by determining the concentration of MB dye at certain intervals after exposing it to the visible light (500 W tungsten halogen lamp with spectral distribution from 400 to 700 nm). The light source was cooled continuously with water cooled jackets so as to maintain ambient temperature. The self-degradation (without catalyst) and the photodegradation of MB using commercial TiO_2_ in visible light were performed for 180 min. It is known that MB self-photolyze under visible light illumination and the percentage efficiency is determined by the utilized light power. It was found that under the present condition; only 21% of MB is photolyzed after 3 h. The self-photolysis has also been reported earlier by Junwang Tang et al. where in around 25% of MB was degraded upon usage of a 300 W Xe arc lamp, λ > 420 nm after 2 h [73,74]. The photocatalytic degradation of commercial TiO_2_ (crystalline anatase, average particle size—0.5 µm, BET surface area 11 m^2^/g, SDFCL, India—Figure S7) was also observed to be minimal which pronounces the inactiveness of commercial TiO_2_ to visible light photocatalytic activity. Interestingly, as seen in Figure 9a, with prolonged exposure to visible light, the concentration of MB dye decreased. The photodegradation rate constant (k), a basic kinetic parameter, was used to quantify the photocatalytic activity of the samples. Reflecting from the previous studies, the experimental data was fitted with the Langmuir-Hinselwood kinetic model ln(C/C_0_) = −kt, where k is the photodegradation rate constant, C is the MB concentration at time *t* and *C*_0_ is the initial MB concentration at time *t* = 0 [75]. It is clearly observed from Figure 9b that under visible light exposure, the ln(C/C_0_) decreases linearly, indicating a first-order reaction. The calculated rate constant was found to be 4.78 × 10^−3^ min^−1^. It could be noted that the photodegradation constant was higher than that of the values reported by Hojamberdiev et al. for SiOC/TiO_2_ nanocomposite [47]. 

**Figure 10:**
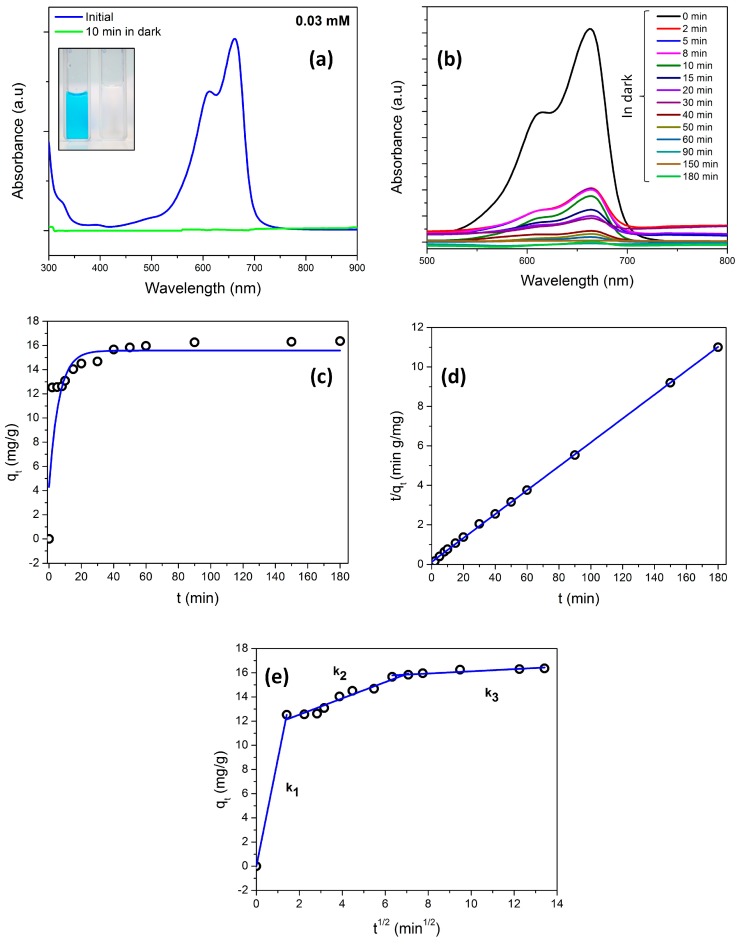
(**a**) UV-vis absorption spectra for 0.03 mM MB with 50 mg of mesoporous nanocomposite; inset: photograph revealing the decolorization of MB; (**b**) UV-vis absorption spectra for 0.03 mM MB with 25 mg of mesoporous nanocomposite; (**c**) pseudo first-order kinetics model; (**d**) pseudo second-order kinetics model; (**e**) intra particle-diffusion kinetic model.

However, for mesoporous nanocomposite, the dye was completely adsorbed by the catalyst within the first 10 min as illustrated in Figure 10a. Hence, in order to evaluate the adsorption kinetics, 25 mg of the catalyst was taken so as to hold back the adsorption process. 

Figure 10b illustrates the UV-vis absorption spectra of the MB solution after the catalyst was added and implies the decrease in concentration of the dye with respect to time. The color of the MB before and after the adsorption is shown in Figure 10a (inset), which clearly indicates complete adsorption. Three different models were used to fit the experimental data to further investigate the adsorption process. The pseudo first-order kinetic equation [76] and the pseudo second-order equation are given by Equations (1) and (2), respectively.

(1) $$q_{t}=q_{e}\left(1-e^{-k_{1}t}\right)$$

(2) $$\frac{t}{q_{t}}=\frac{1}{k_{2}q_{e}^{2}}+\frac{t}{q_{e}}$$

where, *q_e_* and *q_t_* are the amounts of adsorbed MB at equilibrium and time *t*, respectively, whereas *k*_1_ and *k*_2_ denotes the rate constant of pseudo first-order and second-order model, respectively. 

The correlation coefficients of pseudo second-order reaction (R^2^ = 0.999) resulted in a higher value in contrast to the pseudo first-order reaction (R^2^ = 0.735). The *q_e_* value determined from the pseudo first-order reaction was found to be largely deviating from the experimentally obtained equilibrium adsorption capacity (Figure 10c). However, the experimental and fitted values of *q_e_* for pseudo second order reaction were equivalent implying the model is valid (Figure 10d). Since the adsorption kinetics was governed by the pseudo second-order reaction, the overall rate of the adsorption process was controlled by chemisorption [76,77]. The mesoporous sample demonstrated high adsorption capacity (15.2 mg/g) when compared to the macroporous sample (0.16 mg/g). This could be attributed to the high surface area possessed by the mesoporous samples.

The Weber–Morris equation based intra particle-diffusion kinetic model was used to determine the kinetics of steps involved during adsorption.

(3) $$q_{t}=k_{i}t^{0.5}+C$$

where *k_i_* represents the intra-particle diffusion rate constant for adsorption and *C* denotes the boundary layer thickness [78]. Figure 10e clearly depicts the three different intra-particle diffusion rate constants which articulate the rate limiting steps in adsorption process. The fast adsorption process due to the electrostatic interaction between the MB dye molecules and the mesoporous nanocomposites results in a steep slope (*k*_1_ = 8.85); it constitutes the first stage. The gradual diffusion of dye molecules into the mesoporous structure represented by slope *k*_2_ = 0.67 forms the second stage of the adsorption process. Finally, the third stage is characterized by a flat slope (*k*_3_ = 0.09), attributed to the adsorption process at equilibrium.

The high adsorption of MB molecule could be attributed to the high pore volume (0.216 cm^3^/g) as revealed from the pore size analysis. The mesopores result in high surface area which aids the adsorption process. On similar grounds, the adsorption study carried out on carbon-TiO_2_ reported the preferential adsorption of MB molecules (size—1.43 × 0.61 × 1.4 nm) in the micropores and mesopores [65]. The enhanced photocatalytic activity of mesoporous nanocomposites in contrast to the macroporous counterpart can be attributed to the adsorption kinetics. The large surface area aids in the adsorption process by providing enough sites for the catalyst to adsorb dye molecules. The increased amount of adsorption sites as well as the large surface area induced by mesopores is the reason for high adsorption rate in mesoporous structure.

**Figure 11:**
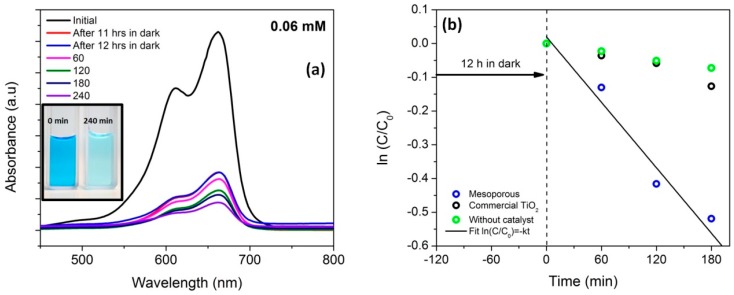
(**a**) UV-vis absorption spectra for 0.06 mM (with 50 mg of mesoporous nanocomposite (inset: photograph revealing the decolorization of MB); (**b**) kinetic analysis of the degradation of MB by mesoporous nanocomposite.

In order to understand the effect of visible light in the photocatalytic activity of mesoporous nanocomposite, a higher concentration of MB solution (0.06 mM) was prepared and kept in dark for 12 h and the solution was exposed to visible light. The change in absorbance spectra as a function of time clearly reveals the decolonization behavior (Figure 11a). The photodegradation constant determined via Langmuir–Hinshelwood kinetic model (Figure 11b) was found to be 3.0 × 10^−3^ min^−1^.

It was found that the degradation constant of macroporous nanocomposite (4.8 × 10^−3^ min^−1^) was higher than the reported values (for 0.03 mM MB solution) as tabulated in Table 1. The structural difference between macro and mesoporous nanocomposite predominantly lies in the porosity aspect since the phases evolved, size and volume percentage of the crystals produced were similar. The mesoporous sample exhibited a BET surface area of 129 m^2^/g and pore volume of 0.22 cm^3^/g in contrast to 0.06 m^2^/g of macroporous sample. It was observed that the concentration of MB chosen (0.03 mM of MB) was completely adsorbed within 10 min. Hence, it was understood that the concentration of MB chosen was not sufficient enough to exhibit photocatalytic activity due to the superior adsorption behavior exhibited by the mesoporous sample. Therefore an intuitive thought process steered to double the concentration (0.06 mM of MB) and subsequently it was observed that the adsorption-desorption equilibrium was attained after 12 h in dark. The photo-degradation constant was then calculated after visible light exposure and has been reported to be 3.0 × 10^−3^ min^−1^ which when compared to the degradation constant of macroporous sample (4.7 × 10^−3^ min^−1^) apparently appears to be low primarily due to the doubled concentration of MB.

### 3.5. Band Gap Measurement

**Figure 12:**
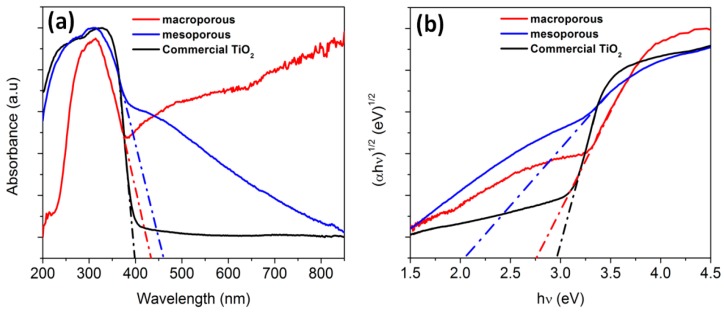
(**a**) UV-vis DRS spectra; (**b**) Tau plot: Namely (αhν)^1/2^ vs. hν of macro/mesoporous nanocomposites and commercial TiO_2_.

Since both the nanocomposites (macroporous and mesoporous) exhibited visible light photocatalytic activity, diffuse reflectance studies were carried out with the aid of UV−vis diffuse reflectance spectroscopy (UV−vis DRS) to comprehend the effect of band gap. The band gap (E_g_) was calculated by the Tauc equation [79,80,81]

(4) $${\left(\alpha h\nu \right)}^{n}=K\left(h\nu -E_{g}\right)$$

where *α* is the absorption coefficient, *hν* is the photo energy, *K* is a constant, and *n* is either 2 for a direct transition or 1/2 for an indirect transition. For TiO_2_ (anatase phase), n is taken as ½ [82]. Figure 12a,b depicts the UV-vis DRS spectra and the band gap energy calculated from the Tauc plot of macro/mesoporous materials and TiO_2_, respectively. The band gap values of macroporous and mesoporous nanocomposites were found to be 2.75 eV and 2.05 eV, respectively. The shift in optical band gap when compared to commercial TiO_2_ is believed to be due to the presence of carbon in the TiO_2_ lattice. The organic titanium precursor (TB) used in this study can effectively be a source of titanium, oxygen and carbon to form carbon doped TiO_2_. When the organic precursor attains a temperature more than its boiling point, it converts to its vapor form. Since the organic precursor contains carbon and owing to the high affinity of carbon towards Ti, diffusion of carbon into TiO_2_ lattice is expected to happen at higher temperatures similar to the observations of Wu et al. on the synthesis of carbon doped TiO_2_ using Ti(OC_4_H_9_)_4_ as precursor [83].

### 3.6. Bonding Characteristics

**Figure 13:**
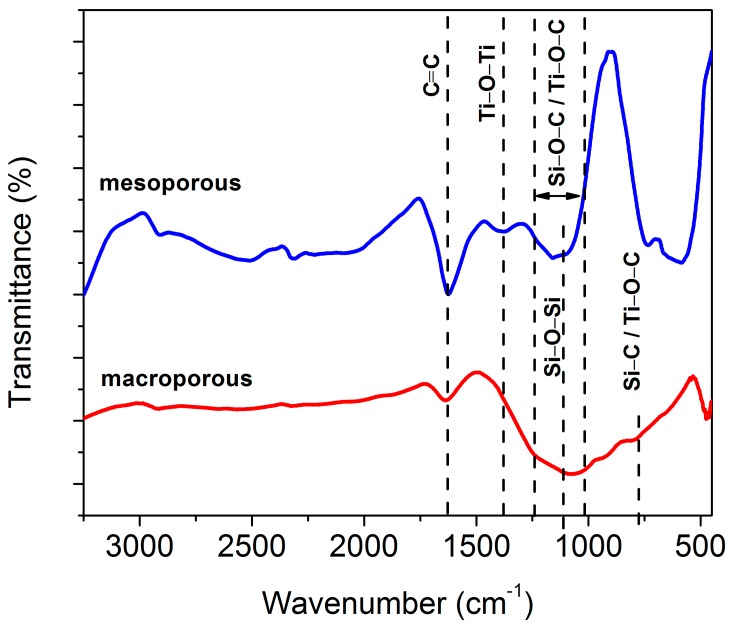
FTIR transmittance spectra of TiO_2_/SiOC nanocomposite indicating the bonding characteristics.

FTIR spectroscopy was done on the macroporous nanocomposites to identify the nature of bonds that compose the materials. It has been compared to the FTIR spectrum of the mesoporous sample. In Figure 13, the broad band in the range of 1020 to 1260 cm^−1^ could be due to the overlapping bands of Si–O–C (1030–1090 cm^−1^) and Si–O–Si (1120 cm^−1^) [84]. The bands located at 796 and 468 cm^−1^ are assigned to Si–C and Ti–O–Ti vibration modes respectively [85]. The absorption bands appearing at 1629 cm^−1^ in the spectra originates from the vibrations of C=C bonds [86]. The broad band in the range 1250–1050 cm^−1^ could also be attributed to the presence of Si–O–Si bonds [87]. The bands at 798 cm^−1^ and 1100 cm^−1^ is assigned to the presence of Ti–O–C bond [88]. The intensity of peak at 1629 cm^−1^ which is a characteristic band for C=C, is significantly higher for the mesoporous nanocomposite. This is a consequence of using carbon templates. In addition, the usage of mesoporous carbon template is expected to be beneficial for the formation of Ti–O–C bonds.

Upon heat-treatment in air, the following reaction is expected to happen:

[CH_3_(H)SiO]_n_ + Ti(OCH_2_CH_2_CH_2_CH_3_)_4_ → [Si(O)CH_2_]_n_ +TiO_2_ + H_2_O ↑ + 4C_4_H_9_OH ↑

**Figure 14:**
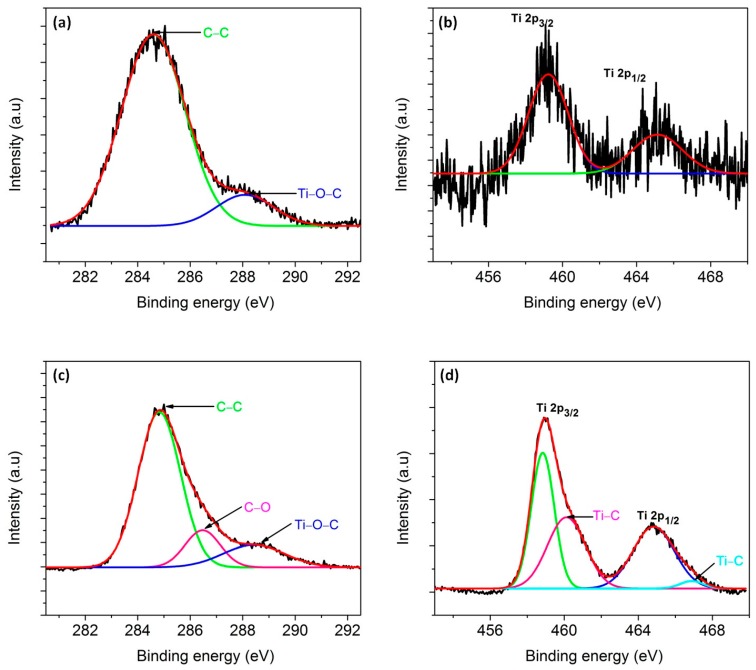
XPS peak deconvolution of C(1s) and Ti(2p): (**a**,**b**) in macroporous and (**c**,**d**) mesoporous materials.

To confirm the presence of carbon in the TiO_2_ lattice, XPS was carried out. The full survey XPS spectra of macroporous and mesoporous materials show the elemental presence of silicon, titanium, oxygen and carbon (Figure S8a). For macroporous nanocomposite (Figure 14b), the peaks centered at 459.2 eV and 465.2 eV corresponds to Ti (2p_3/2_) and Ti (2p_1/2_), respectively and it could be affirmed that Ti present in the nanocomposites is predominantly Ti^4+^. Interestingly, the deconvolution of the Ti peaks of mesoporous nanocomposite resulted in two more peaks other than Ti (2p_3/2_) and Ti (2p_1/2_) at 460.4 and 466.6 eV, which implies the formation of Ti–C bond (Figure 14d) [89]. 

In the case of mesoporous nanocomposite, presence of Ti–C was revealed in XPS (Figure 14d). It is assumed that since the titania precursor was added to SiOC–C composite, the pyrolysis of this mixture could have resulted in the formation of Ti–C bond besides Ti–O–C. The presence of Ti–C bond was also observed by Akhavan et al. when graphene was incorporated in TiO_2_ [89]. The C(1s) spectra of macroporous material could be deconvoluted into two peaks. From Figure 14a,c, the main peak at 284.6 eV is assigned to adventitious elemental carbon whereas the peak at 288.1 eV indicates the presence of Ti–O–C bonds [90]. However, an additional peak was observed in mesoporous nanocomposite at 286.2 eV, which corresponds to the C–O bond. In addition to this, it was observed that the intensity of Ti 2p_3/2_ and Ti 2p_1/2_ of mesoporous nanocomposite was higher in contrast to macroporous nanocomposite. Since XPS is a surface-sensitive quantitative spectroscopic technique, it is assumed that the amount of TiO_2_ nanoparticles exposed on the surface of mesoporous nanocomposite is higher than that of macroporous nanocomposite. The presence of Ti–O–C bonds was observed in both macroporous and mesoporous nanocomposite up on the analysis of C1s peak (Figure 14a,c). XPS studies carried out by Ingo et al. on silica-titania powders assigned an intermediate binding energy value between ~530 eV and ~532 eV to the presence of Si–O–Ti bond [91]. However, the findings also include the breakage of Si–O–Ti bonds upon thermal treatments in air. In the present study, the absence of Si–O–Ti bond was confirmed by the absence of peak at 920 cm^−1^ in the FTIR spectra in both macroporous and mesoporous nanocomposite (Figure 13). 

From the above details, it can be deduced that some of the lattice oxygen atoms may have been substituted by carbon atoms to form a Ti–O–C bond. The introduction of carbon is expected to occur during the heat-treatment/pyrolysis stage. Since Ti atoms have high affinity towards carbon, the diffusion of carbon atoms into the TiO_2_ lattice could have occurred at higher temperatures. We also hypothesize that the use of carbon template aids the doping process. 

The deconvolution of Si peak in macroporous nanocomposite indicates the presence of SiO_2_C_2_ (101.9 eV) and Si–O (103.6 eV) bonds in the matrix (Figure S8b). However, in the case of mesoporous nanocomposites, the deconvolution resulted in the presence of a peak at 103.5 eV only suggesting the presence of Si–O bonds at the surface (Figure S8c). In one of the earliest reported literature by Corriu et al. [92] on the deconvolution of Si 2p peak in XPS of SiOC materials obtained from polysiloxane precursors, the spectrum was proposed to fit using five components with binding energy varying between those of SiO_2_ and SiC as provided below. SiO_4_ (103.5 ± 0.1 eV), SiO_3_C (102.74 ± 0.1 eV), SiO_2_C_2_ (101.9 ± 0.1 eV), SiOC_3_ (101.1 4 ± 0.1 eV), SiC_4_ (100.3 ± 0.1 eV). Hence, it is clear that the binding energies varying from 100.3 to 102.7 eV indicate the presence of SiOC. In addition to this, it was also shown by Dire et al. that the stability of Si–C bonds in polysiloxane networks depends on the metal oxide amount added [93]. It was observed from FTIR results that the high reactivity of titanium atoms towards the Si–C bonds apparently results in the cleavage of Si–C bonds during thermal treatments. The assignment of the binding energies from 100.3 to 102.7 eV indicating the presence of SiOC was also reported by Chandra et al. and Halim et al. [94,95].

### 3.7. Photodegradation Mechanism

The photoexcitation of the produced material in visible light could be attributed to the reduction in the band gap. In Figure 12a, the red shift in the absorption spectra is clearly observed. The red shift observed in this study is attributed to the presence of Ti–O–C/Ti–C bond which is also substantiated by the XPS results. It has been observed that the doping of carbon can occur in two ways, i.e., carbon atoms can either substitute the titanium site or the oxygen site. Kamisaka et al. [96] experimentally observed the visible light response of the catalyst when some of the anion lattice was substituted by carbon. It is believed that during the pyrolysis of initial polymers, there is possibility for some of the oxygen atoms in the TiO_2_ lattice being substituted by the carbon atoms leading to the formation of TiO_2−2x_C_x_${\left[V_{O_{2}}^{¨}\right]}_{x}$, where ${\left[V_{O_{2}}^{¨}\right]}_{x}$ is the oxide ion vacancy. Electron spin resonance (ESR) investigation was further carried out at room temperature to confirm the presence of oxygen vacancies. Both the macroporous and mesoporous nanocomposites exhibited EPR signals at about g = 2.00 as illustrated in Figure S9. The g value clearly indicates the single electron trapped oxygen vacancy [97].

**Figure 15:**
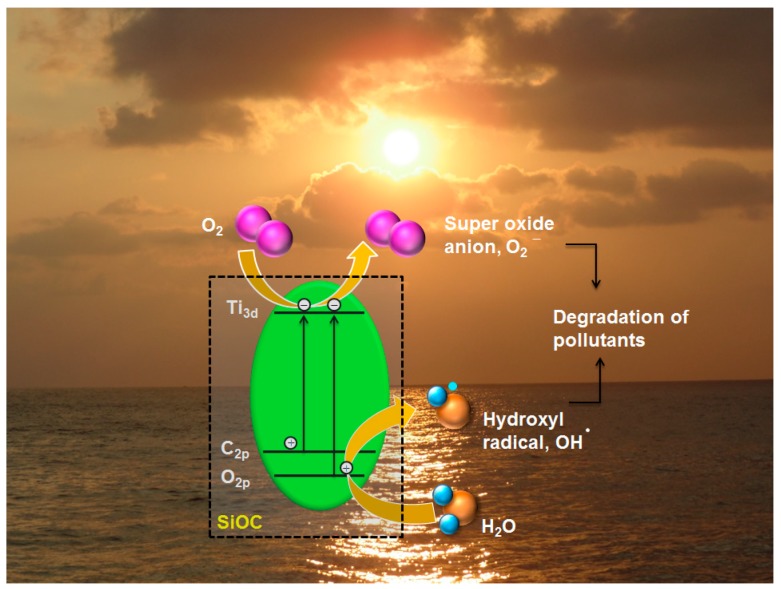
Schematic representation of band gap reduction mechanism and degradation process.

The binding energy of the carbide (2p) band is ~3 eV less when compared to that of O^2−^ (2p) band. Hence, it is believed that the substitution of C^4−^ ion should reduce the band gap as the C^4−^ (2p) band lies above the O^2−^ (2p) band and the optical transitions happens between the C 2p_π_ to Ti d_xy_ instead of O 2p_π_ to Ti d_xy_ (Figure 15) [98]. 

In general, low concentration of dye can only be removed by the photocatalytic degradation process. To treat a highly concentrated dye solution, materials that can contribute to the combinatorial effect of adsorption and photocatalysis is designed and mesoporosity serves that purpose. The photodegradation process is expected to be occurring in two steps. The first step involves the adsorption of MB molecules onto the samples (both in macro and mesoporous nanocomposite), while colossal adsorption occurs in the mesoporous couterparts. Subsequently, upon the illumination of visible light, the active TiO_2_ nanocrystals embedded in SiOC matrix gets excited to generate hydroxyl and superoxide radicals. This eventually will lead to the decomposition of MB molecules. The adsorption process only governs the kinetics of the degradation mechanism. 

We also hypothesize the effect of heterojunction formation between amorphous SiOC and crystalline TiO_2_ to enhance the photocatalytic effect. The heterojunctions formed between the above is expected to reduce the recombination rate of photogenerated electron-hole pair. Recently, a study has been carried out on the amorphous phase of polymer derived silicon oxycarbide and has reported a band gap value of 2.5 eV. In addition to this, for amorphous SiOCN, the dependence of band gap on the pyrolysis temperature has been studied by Wang et al. [99] and concluded that the band gap decreases with increase in pyrolysis temperature. 

**Figure 16:**
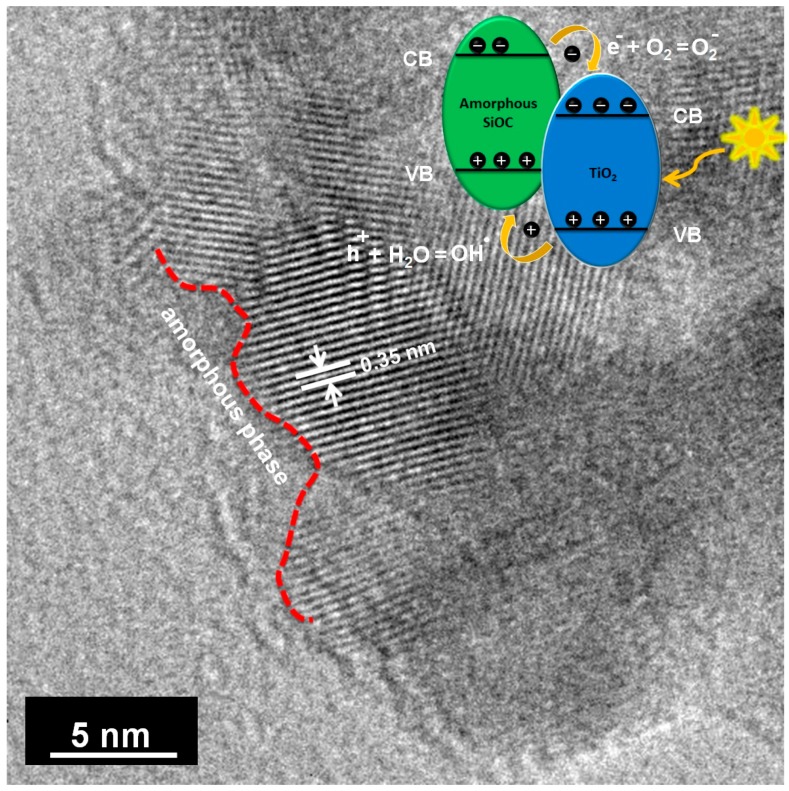
Schematic diagram of the heterojunction formation between TiO_2_ and SiOC.

As inferred from the work of Zou et al. [100] it is believed that the conduction and valence band potential of SiOC is higher than that of TiO_2_. Thus the photogenerated electrons on the surface of SiOC will be transferred to the conduction band of TiO_2_, whereas the holes in the valence band of TiO_2_ transports to the valence band of SiOC, thereby reducing the recombination rate of the electron-hole pair. A schematic representation of the same is shown in Figure 16.

## 4. Conclusions

In situ TiO_2_/SiOC nanocomposites were successfully prepared through a precursor derived route. The heat-treatment/pyrolysis temperature as well as the volume percentage of the starting precursor governs the crystallization of TiO_2_ in an amorphous SiOC matrix. The high surface area imparted by the mesoporous structure increased the adsorption capacity of mesoporous nanocomposites. The synthesized nanocomposites exhibited a reduction in band gap when compared to pure titania and considerable photocatalytic activity in the visible light. The formation of Ti–O–C/Ti–C bonds (reduction in band gap) and the heterojunction formed between the crystalline and amorphous phase (reduced recombination rate) is believed to enhance the photocatalytic activity.

## Figures and Tables

**Table 1:** Comparison of photodegradation constants of various photocatalysts.

Material	K (10^−3^ × min^−1^)
SiOC	1.3 [47] (0.03 mM of MB)
SiOC/TiO_2_	2.2 [47] (0.03 mM of MB)
SiOC/N-doped TiO_2_	3.4 [47] (0.03 mM of MB)
Macroporous TiO_2_/SiOC	4.8 [present work] (0.03 mM of MB)
Mesoporous TiO_2_/SiOC	3.0 [present work] (0.06 mM of MB)

## Supplementary Materials

The following are available online at http://www.mdpi.com/1996-1944/11/3/362/s1, Figure S1: TGA curve exhibiting the mass loss as a function of temperature for 50 vol % PHMS-TB mixture, Figure S2: X-ray diffractograms of 50 vol % SiOC–TiO_2_ as a function of pyrolysis temperature (Anatase-JCPDS card No. 20-2242, Rutile-JCPDS card No. 21-1276, Silica-JCPDS card No. 89-3607), Figure S3: X-ray diffractogram of SiOC–TiO_2_ as a function of varying vol % of titanium (n-tetrabutoxide). Pyrolysis was carried out at 1200 °C, Figure S4: Rietveld refinement of (a) macroporous and (b) mesoporous nanocomposite using MAUD software, Figure S5: (a) SEM and (b) TEM micrographs of mesoporous carbon, Figure S6: SAED pattern of (a) macroporous and (b) mesoporous nanocomposites, Figure S7: (a) X-ray diffractogram revealing the presence of crystalline anatase phase (b) particle size distribution histogram and (c) Nitrogen adsorption-desorption curve of commercial TiO_2_, Figure S8: XPS (a) full survey spectra indicating the presence of Si, Ti, O and C (b) Si2p macroporous and (c) Si2p mesoporous nanocomposites, Figure S9: EPR spectra of macroporous and mesoporous SiOC/TiO_2_ clearly indicating the presence of vacancies, Table S1: Comparison of Raman vibrational modes of macroporous and mesoporous nanocomposites with single crystal anatase.
