# Supplementary material for: Novel Precursor-Derived Meso-/Macroporous TiO2/SiOC Nanocomposites with Highly Stable Anatase Nanophase Providing Visible Light Photocatalytic Activity and Superior Adsorption of Organic Dyes
Source: Materials (Basel). 2018 Mar 1;11(3):362. doi: 10.3390/ma11030362 (PMC5872941; doi:10.3390/ma11030362)
Supplement: Supplementary file 1 [file materials-11-00362-s001.docx]

**Supplementary data**

Eranezhuth Wasan Awin ^1^, Abhijeet Lale ^2^, Kollamala Chellappan Nair Hari Kumar ^1^, Umit Bilge Demirci ^3^, Samuel Bernard ^2^ and Ravi Kumar ^1,^*

^1^ Laboratory for High Performance Ceramics, Department of Metallurgical and Materials Engineering, Indian Institute of Technology Madras (IIT Madras), Chennai 600036, India;
eranezhuth@gmail.com (E.W.A.); kchkumar@iitm.ac.in (K.C.H.K.)

^2^ Science des Procédés Céramiques et de Traitements de Surface (SPCTS), UMR CNRS 7315, Centre Européen de la Céramique, 12 rue Atlantis, 87068 Limoges CEDEX, France; abhijeet.lale@unilim.fr (A.L.); samuel.bernard@unilim.fr (S.B.)

^3^ IEM (Institut Europeen des Membranes), UMR 5635 (CNRS-ENSCM-UM), Universite Montpellier, Place E. Bataillon, F-34095 Montpellier, France; Umit.Demirci@iemm.univ-montp2.fr

***** Correspondence: [nvrk@iitm.ac.in](mailto:nvrk@iitm.ac.in)


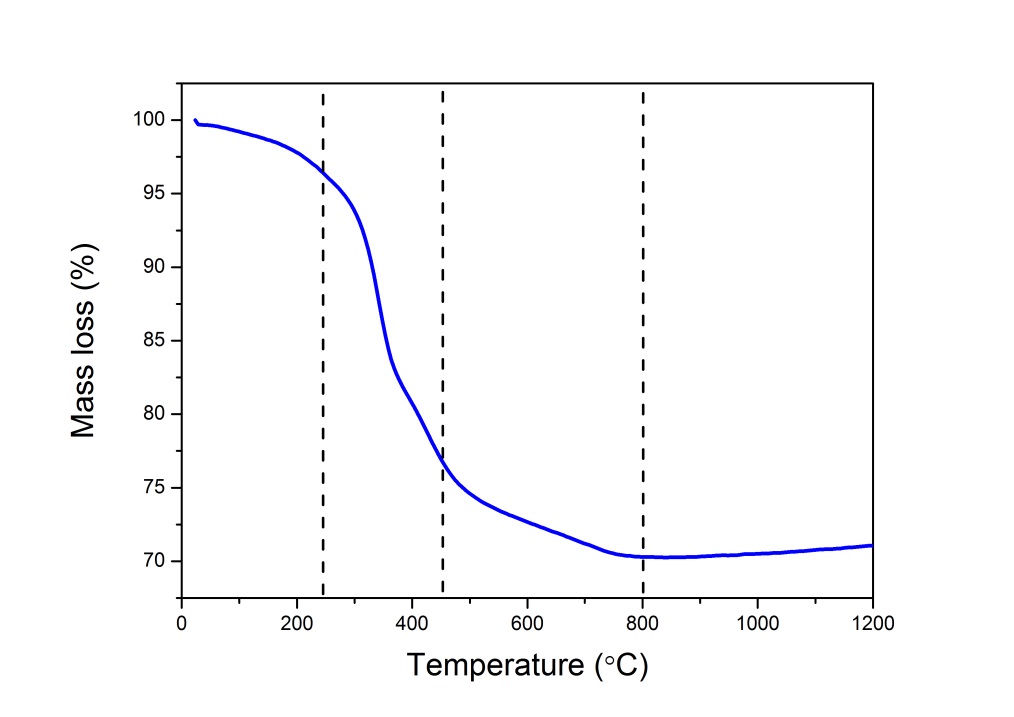


**Figure S1.** TGA curve exhibiting the mass loss as a function of temperature for 50 vol % PHMS-TB mixture.


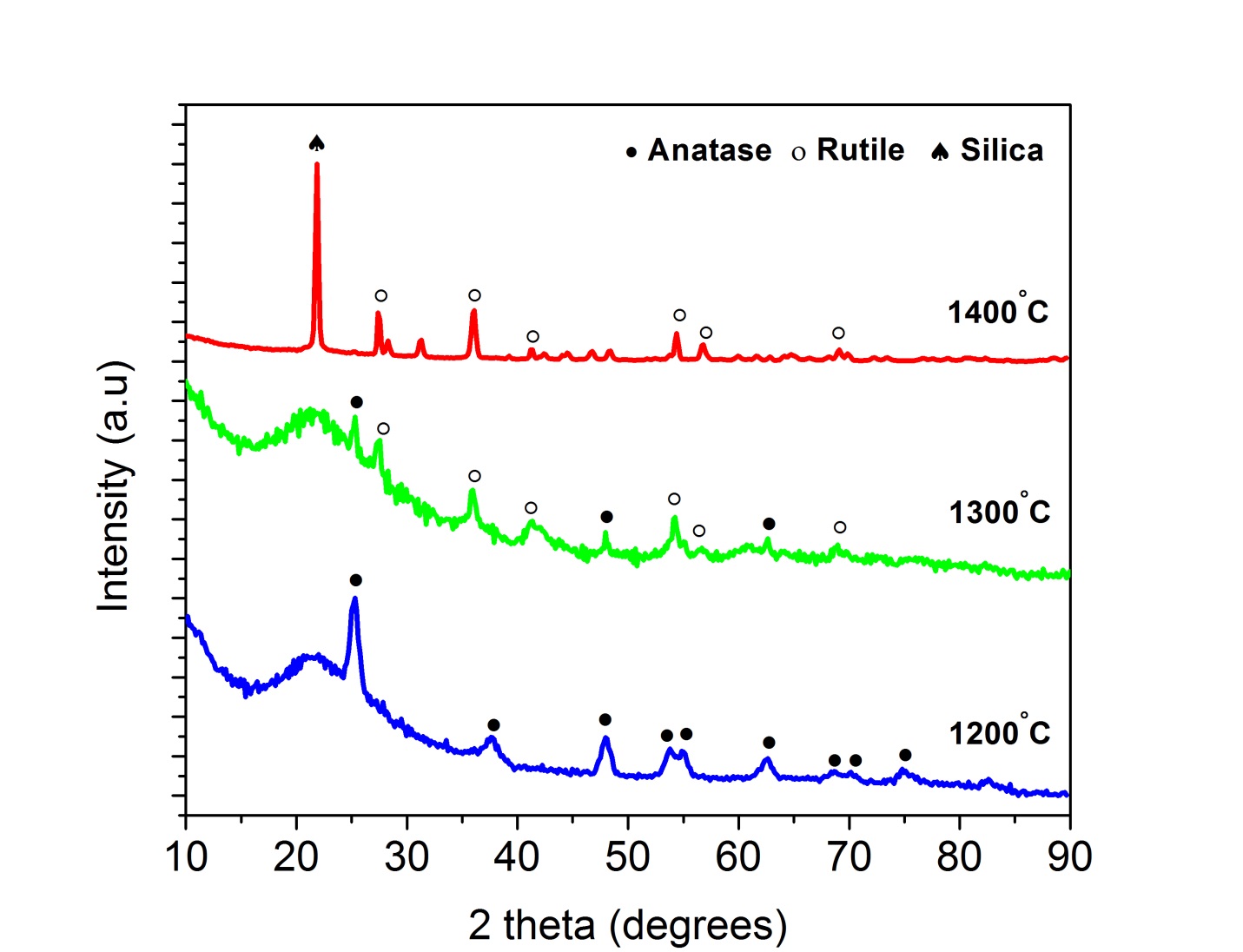


**Figure S2.** X-ray diffractograms of 50 vol % SiOC–TiO_2_ as a function of pyrolysis temperature (Anatase - JCPDS card No. 20-2242, Rutile – JCPDS card No. 21-1276, Silica - JCPDS card No. 89-3607)

**
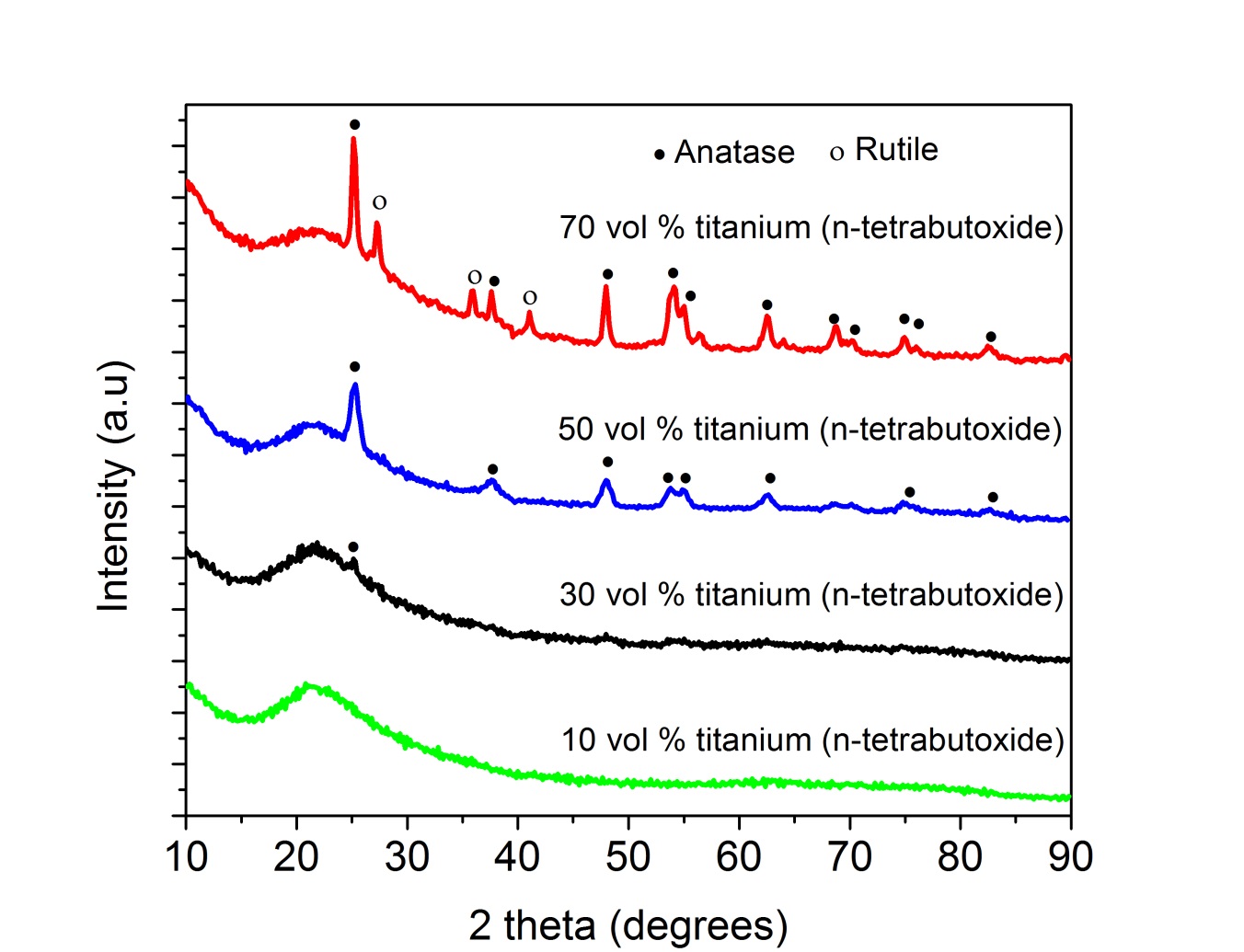
**

**Figure S3.** X-ray diffractogram of SiOC–TiO_2_ as a function of varying vol % of titanium (n-tetrabutoxide). Pyrolysis was carried out at 1200°C.


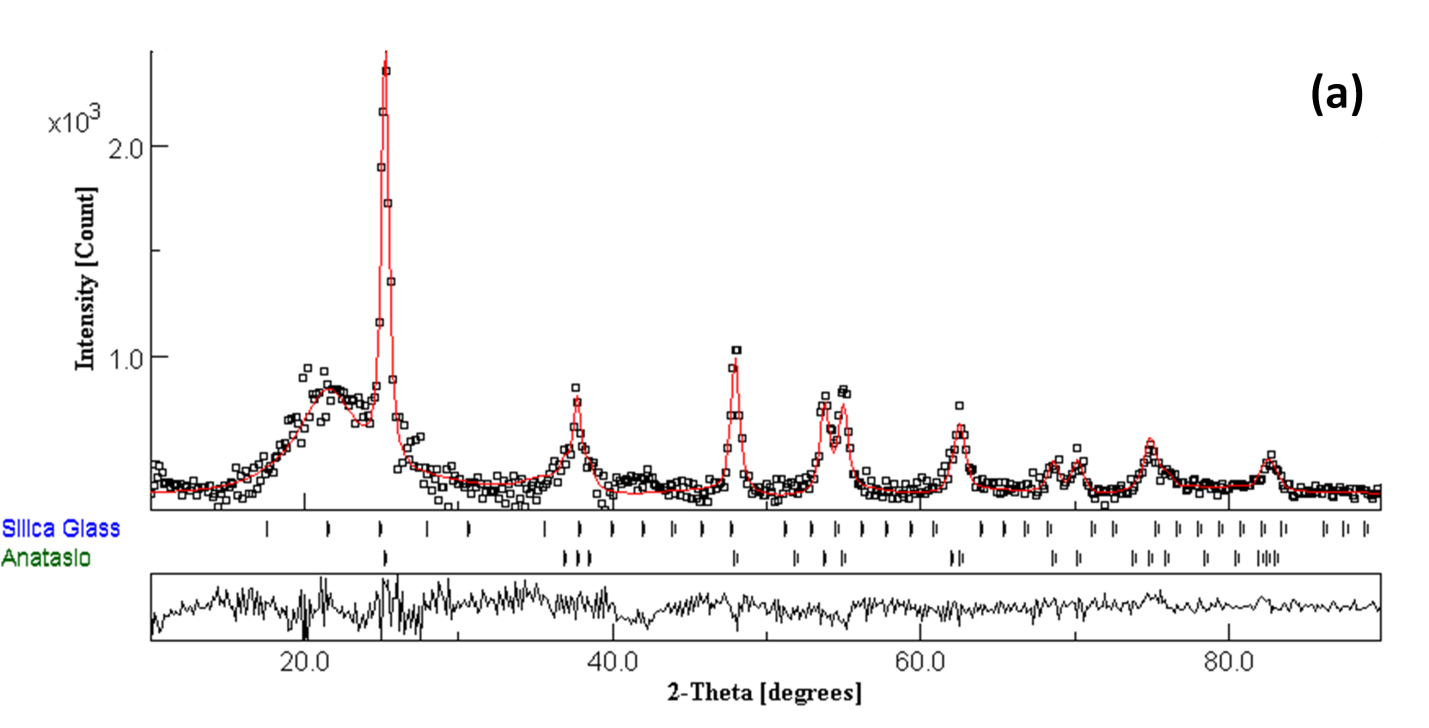


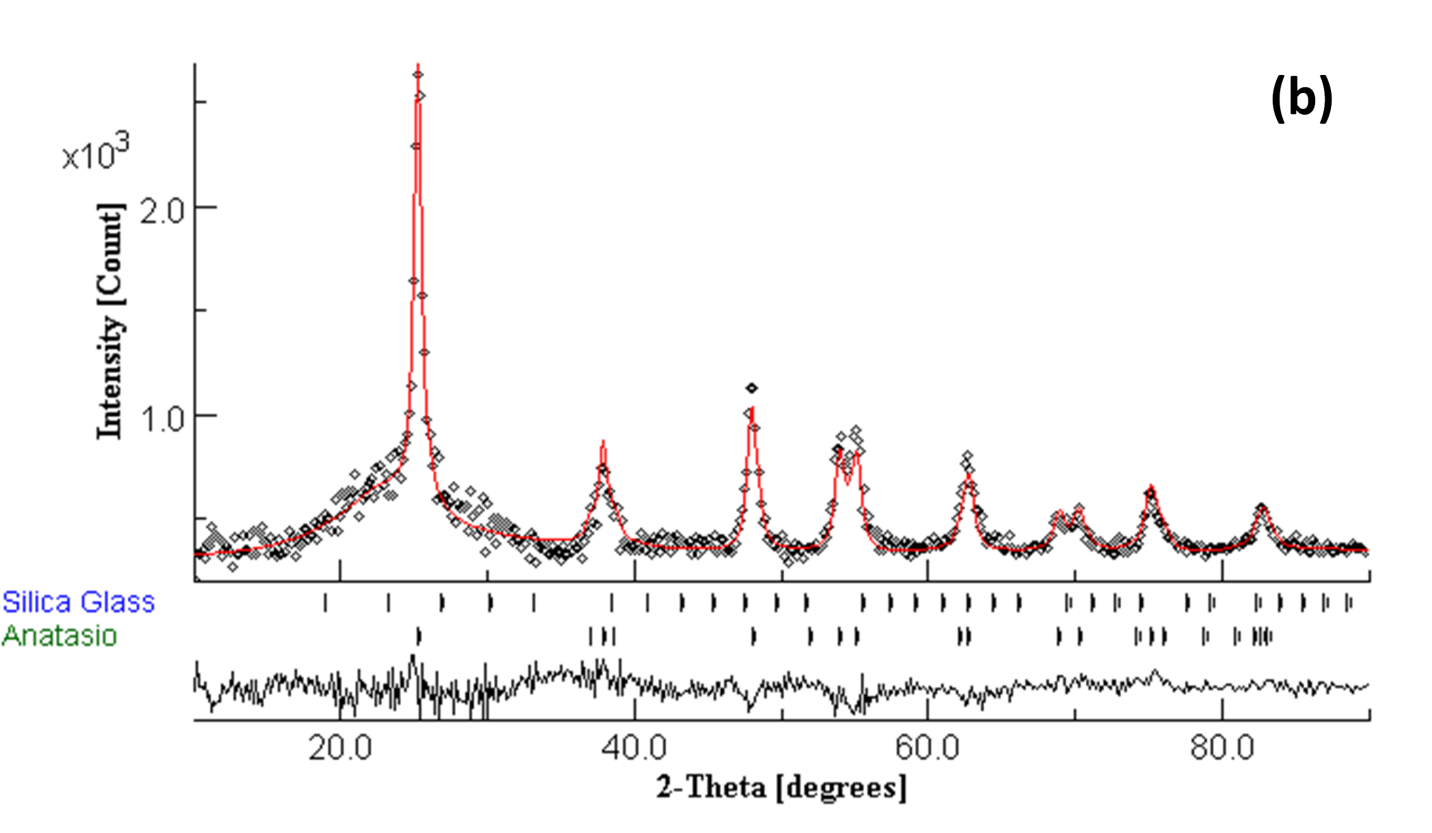


**Figure S4.** Rietveld refinement of (a) macroporous and (b) mesoporous nanocomposite using MAUD software.


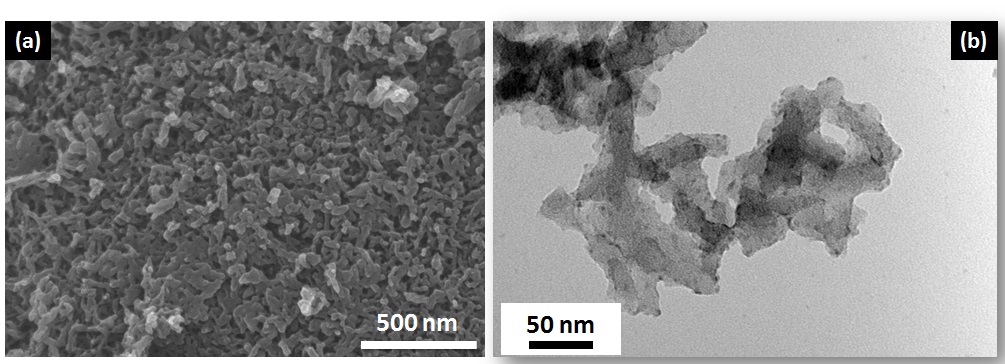


**Figure S5.** (a) SEM and (b) TEM micrographs of mesoporous carbon

**Table S1.** Comparison of Raman vibrational modes of macroporous and mesoporous nanocomposites with single crystal anatase.

| **Samples** | **E_g_ (cm^-1^)** | **E_g_ (cm^-1^)** | **B_1g_ (cm^-1^)** | **A_1g_+B_1g_ (cm^-1^)** | **E_g_ (cm^-1^)** |
| --- | --- | --- | --- | --- | --- |
| Single crystal anatase^1^ | 144 | 197 | 399 | 519 | 639 |
| macroporous | 145 | 198 | 396 | 509 | 634 |
| mesoporous | 151 | 200 | 395 | 511 | 630 |


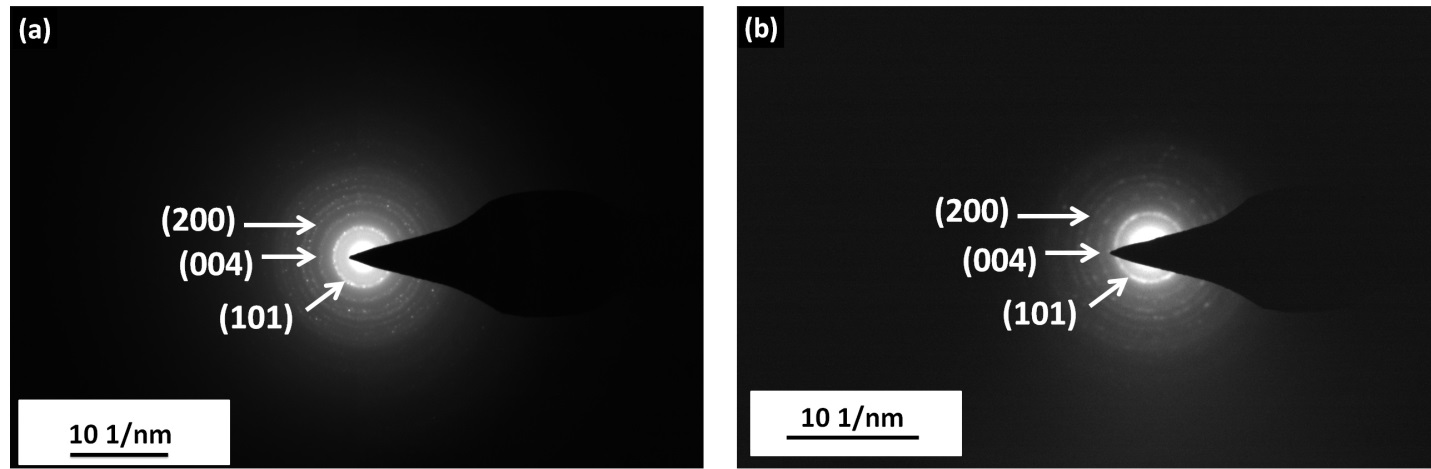


**Figure S6.** SAED pattern of (a) macroporous and (b) mesoporous nanocomposites


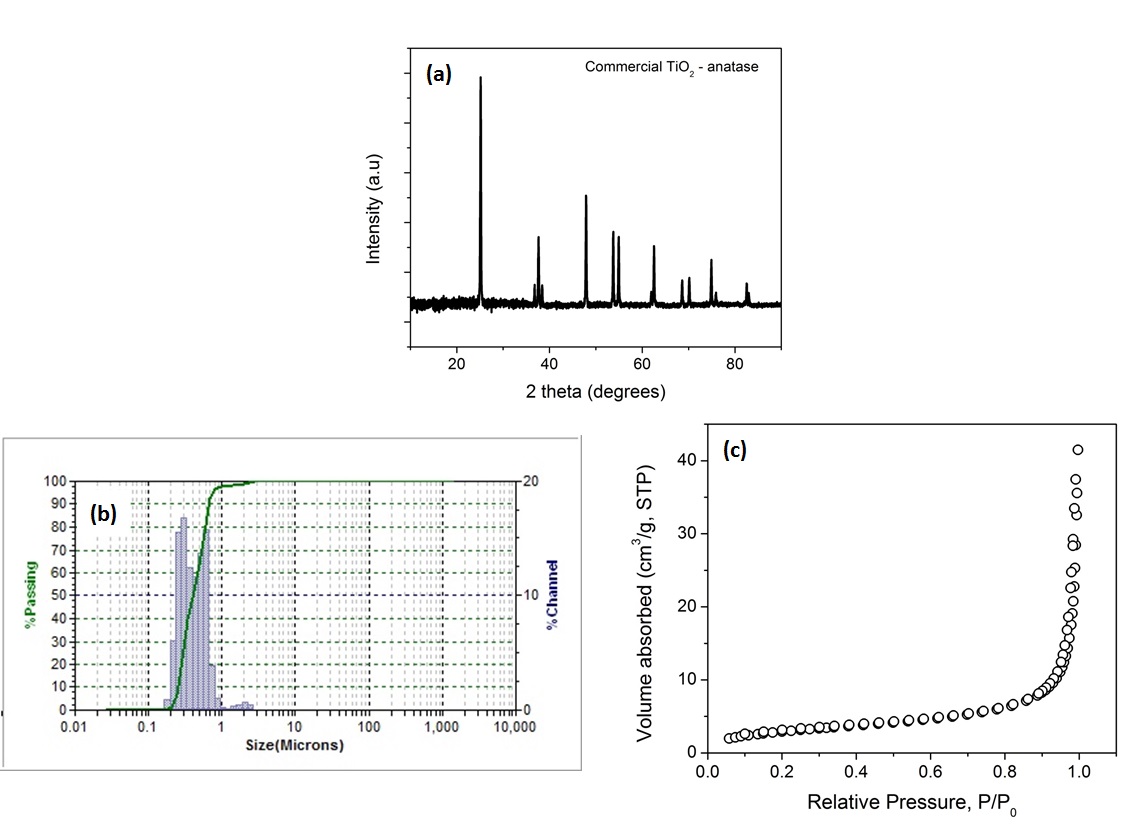


**Figure S7** (a) X-ray diffractogram revealing the presence of crystalline anatase phase (b) particle size distribution histogram and (c) Nitrogen adsorption-desorption curve of commercial TiO_2_


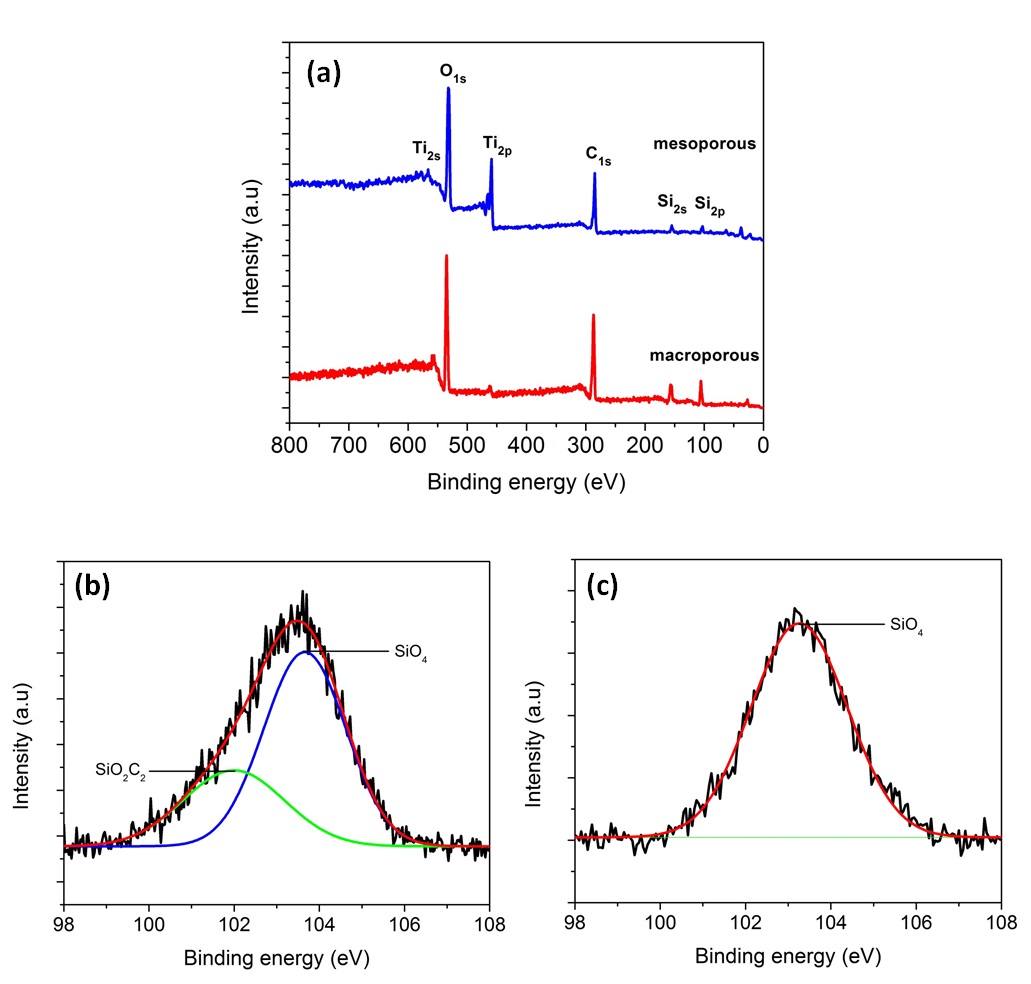


**Figure S8** XPS (a) full survey spectra indicating the presence of Si, Ti, O and C (b) Si_2p_ macroporous and (c) Si_2p_ mesoporous nanocomposites.


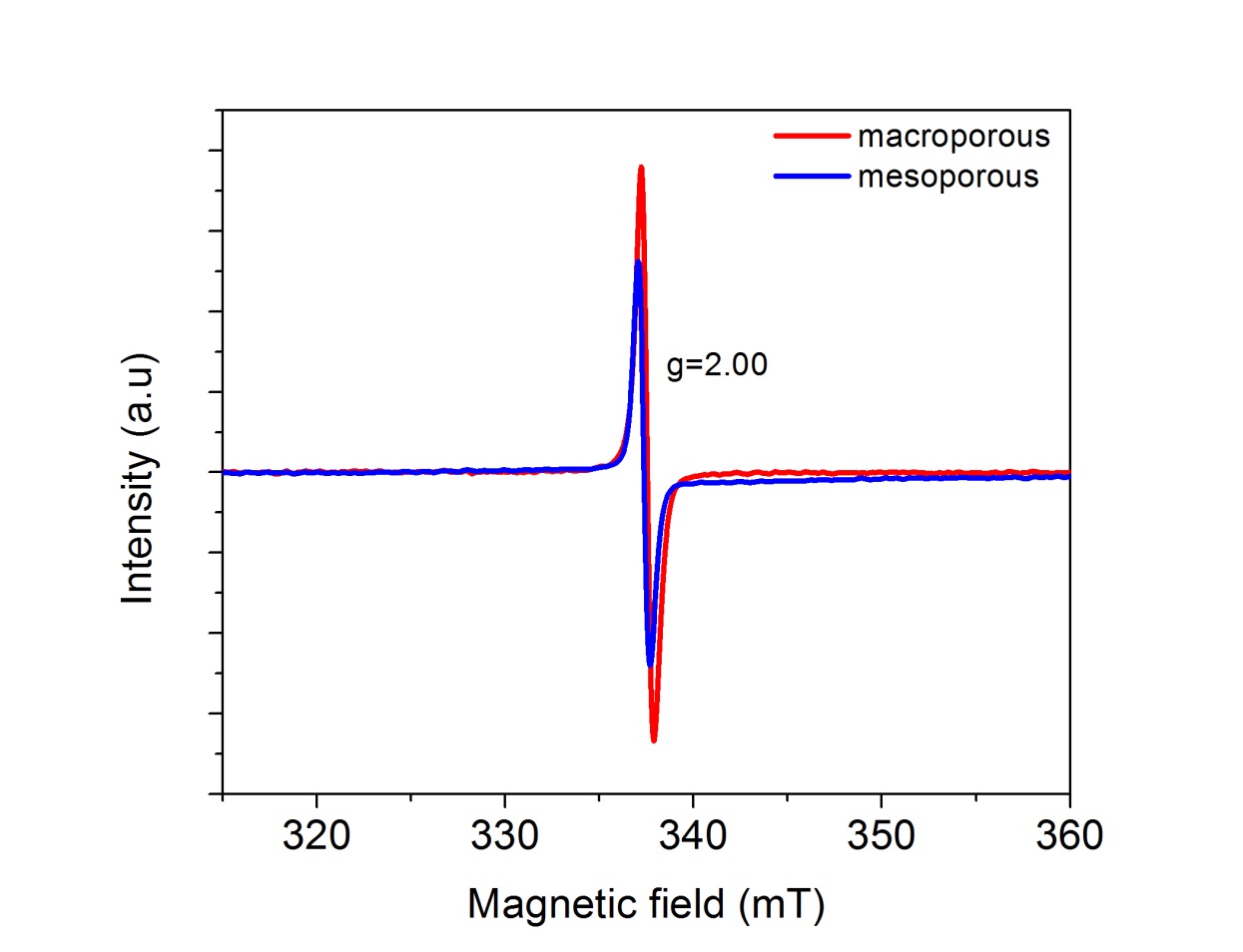


**Figure S9.** EPR spectra of macroporous and mesoporous SiOC/TiO_2_ clearly indicating the presence of vacancies.

**Reference:**

[1] X. Shao, W. Lu, R. Zhang and F. Pan, *Sci. Rep*., 2013, 3, 1–9.
